# Zamzam Water Ameliorates Gentamicin-Induced Testicular Toxicity in a Rat Model via Targeting Sperm Parameters, Testicular Tissue Oxidative Insult, Inflammation, Apoptosis, and Pituitary-Gonadal Axis

**DOI:** 10.3390/toxics11010002

**Published:** 2022-12-20

**Authors:** Medhat Taha, Sara T. Elazab, Abdullah A. Saati, Gomaa S. Ahmed, Tourki A. S. Baokbah, Khaled Fathy, Ibrahim El-Shenbaby, Omer Abdelbagi, Mahmoud A. E. Hassan, Mohie Mahmoud Ibrahim, Alaa. M. Badawy

**Affiliations:** 1 Department of Anatomy and Embryology, Faculty of Medicine, Mansoura University, Mansoura 35516, Egypt; 2 Department of Anatomy, Al-Qunfudah Medical College, Umm Al-Qura University, Al-Qunfudhah 28814, Saudi Arabia; 3 Department of Pharmacology, Faculty of Veterinary Medicine, Mansoura University, Mansoura 35516, Egypt; 4 Department of Community Medicine and Pilgrims Healthcare, Faculty of Medicine, Umm Al-Qura University, Makkah 24382, Saudi Arabia; 5 Department of Dermatology, Venereology and Andrology, Faculty of Medicine, Assiut University, Assiut 71515, Egypt; 6 Department of Medical Emergency Services, College of Health Sciences-AlQunfudah, Umm Al-Qura University, Al-Qunfudah 28814, Saudi Arabia; 7 Electron Microscopy Unit, Mansura University, Mansoura 35516, Egypt; 8 Clinical Pharmacology Department, Faculty of Medicine, Mansoura University, Mansoura 35516, Egypt; 9 Department of Pathology, Qunfudah Faculty of Medicine, Umm-Al-Qura University, Makka 24382, Saudi Arabia; 10 Agriculture Research Center, Animal Production Research Institute (APRI), Ministry of Agriculture, Dokki, Giza 12619, Egypt

**Keywords:** infertility, testicular damage, gentamicin, zamzam water, alkaline water, antioxidant

## Abstract

Gentamicin is considered one of the most typical causes of testicular damage. Oxidative stress is a significant contributor to testicular tissue damage. Zamzam water (alkaline in nature) has an antioxidant effect. The purpose of this study was to assess the potential palliative effect of Zamzam water against gentamicin-induced testicular damage. Thirty Rats were separated into three groups, each with ten rats, as follows: The Control received only normal saline. The gentamicin group received 100 mg/kg/day of gentamicin intraperitoneally for six days from day 15 to the end of the experiment. The gentamicin +Zamzam Water group received a dose of gentamicin 100 mg/kg/day intraperitoneally with Zamzam water as their sole source of drinking from day one to day 21. Hormonal assay in serum, histological, immunohistochemical, and ultrastructural examination of testicular tissue with a molecular study were obtained. Pretreatment with Zamzam water significantly *p* < 0.001 increased serum levels of testosterone, FSH, and LH, as well as the percentage of sperm motility and progressive motility. It also upregulated SOD, CAT, GPx enzymatic activity, gene expression of Nrf2/HO-1, and immunoexpression of PCNA. While the percentage of dead sperm and abnormal sperm, immunoexpression of NFκB, Caspase 3, inflammatory cytokines TNFα, IL-1β, IL-6, and MDA levels significantly (*p* < 0.001) declined with histological improvement. It was concluded that Zamzam water as alkaline water possesses antioxidant, anti-inflammatory, and antiapoptotic effects against gentamicin-induced testicular toxicity in vivo.

## 1. Introduction

One of the most exciting health problems is infertility, with 12% of couples facing the problem of infertility, while 50% contribute to male infertility [1]. Many environmental factors are involved in male infertility, such as environmental toxins and xenobiotics, which affect the process of spermatogenesis and the production of mature motile spermatozoa [2]. 

Gentamicin is considered one of the commonest aminoglycosides widely prescribed for Gram-negative bacteria eradication [3]. The everyday use of aminoglycosides may be due to the resistance to other antibiotics families [4]; unfortunately, treatment with gentamicin is accompanied by testicular toxicity, so giving limitations to its use [5]. Many of the previous works attributed that treatment with 100 mg of gentamicin for 10 days seriously affects sperm motility and affects the testicular tissue [6]. An essential factor in the etiology of gentamicin-induced testicular damage is oxidative stress [7], as it affects the function of the sperm leading to reproductive disorder and subsequent male infertility [8]. 

Oxidative stress is considered one of the significant attributing factors for male infertility and affection of reproductive function as it was found to be harmful not only to germ cell development but also to the function of spermatozoa [9]. The plasma membrane of the sperm and testicular tissue are rich with polyunsaturated fatty acids, which provide the fluidity of the sperm and is thought to be essential for the capacitation step of the fertilization processes [10]. Unfortunately, polyunsaturated fatty acids are more liable to lipid peroxidation and ROS overproduction [11]; between 25% to 40% of infertile men's semen analysis shows a high level of ROS [12]. The alternation of the membrane integrity with ROS overproduction impaired the viability and motility of the sperm [13]; therefore, much research has examined several antioxidants against gentamicin-induced renal toxicity [14].

Alkaline waters were involved in combating oxidative stress in chronic renal disease patients [15] and improving the aging process, as oxidative stress is considered its major contributor [16]. At the same time, it improves the glycemic index in STZ-induced diabetic rats, but until now, its role has not been fully understood [17]. The beneficial effect of alkaline water is its alkaline property and its composition of trace elements such as magnesium [18]. The fear of contamination should be considered; in this context, its hydrophobic character offers another significant effect [19].

Zamzam water is extracted from the Zamzam well, which is present in the Holy Mosque east of Kaba in Makkah, Saudi Arabia. The Zamzam well has a depth of 30.5 m underground with a diameter of 1.08 to 2.66 m. The advantages of Zamzam water from other waters are that it is free from contamination with bacteria or pathogens and also it does not change its color and taste, or undergo molding [20] or biological growth of vegetation in the wells as a result of the growth of algae, which would normally change the taste and color of the water, however, this biological action does not happen in the Zamzam water well [21]. The chemical analysis of Zamzam water has revealed that it has no specific smell or odor, but has a clear taste; the PH of Zamzam water is alkaline in its nature at 7.9–8.0, and the minerals are concentrated as follows: 96 mg/L of ca^++^, 133 mg/L of Na^+^, 0.72 mg/L of fluoride, 43.3 mg/L of K^+^, 38.88 mg/L of magnesium, 163.3 mg/L of chloride, 124.0 mg/L of sulfate, 124.8 mg/L of nitrate, and 195.4 mg/L of bicarbonate. Zamzam water’s significant antioxidant qualities are a result of its alkaline nature, which is a result of the variety in its mineral content [22]. Bamosa et al. [23] reported that type 2 diabetic patients who were supplemented with Zamzam water for two months showed the elevation of the antioxidant parameters with downregulation of the level HbA1c. In addition, previous work showed that treatment of gentamicin-induced stress rats was associated with upregulation of the antioxidant capacity [23]. Several previous works have explored the beneficial effect of Zamzam water against diabetes, nephrotoxicity, hepatotoxicity, and stress; these studies were carried out either on rat models or humans [24,25]. 

Prior research has reported an enhancement in human sperm motility and capacitation after culturing in an alkaline nutrition solution at pH 7.2 and 8.2. In addition, the report pointed out that an acidic environment caused a reduction in sperm movement and capacitation as a consequence of suppressing Na^+^/K^+^-ATPase activity, which in turn may lead to male infertility [26]. Furthermore, LG et al. [27] noticed that the semen pH was less than 7.2 in individuals suffering from oligospermia and/or asthenospermia. What is more, in turkey and quail species, the proportion of motile sperm was accelerated at alkaline pH [28]. The alkaline nature of Zamzam water, with its prominent antioxidant features, motivated us to hypothesize that this water may alleviate gentamicin-induced testicular injury. Thus, this study aimed to examine Zamzam water’s anti-inflammatory, antioxidant and antiapoptotic properties against testicular damage induced by gentamycin.

## 2. Materials and Methods

### 2.1. Animals

Thirty adult male rats weighing 200–250 g were purchased from the faculty of veterinary medicine, Zagazig University, Egypt. They were kept for two weeks before the study to acclimatize to the experiment. These rats were housed in three plastic cages at a room temperature of 22–24 °C and 50–60% humidity, with a 12/12 h day and night cycle. A regular pellet feed and fresh tap water was offered. All our research protocols adhere to the recommendation for using laboratory animals published by the US national institute of health (NIH No. 85–23, revised 2011) and were accepted by the Mansoura University Animal Care and Use Committee with ethical approval code MU-ACUC (VM.R.22.10.10).

### 2.2. Experimental Groups

Rats were separated into three groups, each with ten rats, as follows; the Control group (Group I): rats in this group received only normal saline by oral route at a rate of 10 mg/kg/day for 21 days. The gentamicin group (Group II): rats in this group received 100 mg/kg/day of gentamicin (Memphis Pharma Production, Egypt) intraperitoneally for six days; this gentamicin dosage has been examined in earlier studies [5]. It was found that treating the rats with this dose exhibited multiple organ damage. Group III (gentamicin + Zamzam water group): Rats of this group received a dose of 100 mg/kg/day intraperitoneally for six days with Zamzam water in a dose of 100 mL/24 h/cage as their sole source of drinking water from day one of the experiments to day 21 the amount of Zamzam water according to previous research [29]. The experiment was carried out in the department of pharmacology, Veterinary Medicine collage, University of Mansoura, Egypt.

### 2.3. Assessment of the Weight of the Testis and Specimen Collection

Rats from all groups were weighed at the end of the experiment, and then they were given anesthesia with 5 mL/kg of xylazine and 50 mg/kg of ketamine. Blood samples were then taken from a retro-orbital vein using a 5-gauge syringe, centrifuged for 10 min at 3000× g, and the serum was removed and stored at −80 °C for hormonal assay. Rats were killed by aortic dissection, and both testes were removed and weighted; the cauda epididymis was cut from both testes and weighted. The right testes of all groups were cut into three slices. The first slice was fixed in Bouin’s solution for histological investigation using H&E and PAS staining. In contrast, the second was fixed with 10% formalin for immunohistochemical studies, and the third slice was fixed with 2.5% glutaraldehyde for ultra-structure examination. The left testes of the three groups were washed up with phosphate saline solution and homogenized with phosphate buffer saline (pH~7–7.2), then centrifuged for 15 min at 20,000× *g* using the (Glas-Col: Veron Hills, IL, USA) homogenizer.

### 2.4. Assessment of Sperm Parameters (Motility and Morphology)

For analysis of spermatozoa, samples of semen were taken from the cauda epididymis; for assessment of progressive motility of the sperm, the semen sample was diluted with 0.9% NaCl; for determination of the progressive motility of the sperm, we examined three microscopic fields for each sample of semen by using a phase-contrast microscope (Leica DM500, Leica, Mikrosysteme, Vertrieb GmbH) at 37 °C temperature. The percentage of unstained live sperm or stained dead sperm and abnormal spermatozoa can be counted in the same semen field (300 sperm cells/sample) by staining it with a mixture of 10% nigrosine and 5% eosin stains and examining it with contrast microscopy at ×400 magnification.

### 2.5. Hormonal Assay 

Rats’ testosterone ELISA kits from Mybiosource Southern California, San Diego (US) (Catalog No: MBS 766199), Rats FSH ELISA kits (Catalog No.CSB-E06869r), and kits of LH ELISA (Catalogue No. CSB-E12654r) were used to measure the levels of serum testosterone, follicular stimulating hormone, and luteinizing hormone according to the manufacture. 

### 2.6. Oxidative Stress Measurement 

Lipid peroxidation markers like malondialdehyde (MDA) and antioxidant enzymes like CAT, GPx, and SOD were measured in the homogenate of the testicular tissue using the following Kits, respectively: MD 2529, CA2517, GP 2524, and SD 2521. All the kits mentioned above were purchased from Biodiagnostic, Giza, Egypt.

### 2.7. Microscopic Examination 

Some of the right tests from different groups were put in Bouin’s fixative for 24 h. It is then treated with an increasing concentration of alcohol, followed by xylene, and paraffin immersion. Five-μm sections from paraffin blocks were deparaffinized, rehydrated, and then stained with hematoxylin and eosin for light microscopy study of seminiferous tubules. The pathologist blindly examined various sections of testes fitting to Johnson’s counting system for testicular tissue Table 1 [30]. Acquired pictures were evaluated using software J image (type 1, 50b. US) [31]. From each animal, 30 tubules were examined and scored from 0 to 10 depending on the presence or absence of seminiferous tubules, germ cells such as sperm, spermatids, spermatocytes, spermatogonia, and somatic cells. The highest score indicates spermatogenesis, and the lowest score indicates hypospermatogenesis. A score of 10 represents the highest degree of maturation of the seminiferous tubular epithelium with maximal activation of the tubules, and a score of 0 represents complete inactivation of the seminiferous tubules, indicating that the seminiferous tubular epithelium is the least mature [32]. To investigate common carbohydrates in tubules, 5-μm sections were stained with PAS stain. [33].

### 2.8. Immunohistochemical Assay of Caspes3, NF-kβ and PCNA

For immunohistochemical examination, 5μm sections from the paraffin block were dewaxed and dehydrated by the different scales of alcohol. For antigen retrieval, we used 0.05 mole of a citrate buffer solution with pH 6.8. To block protein, we applied 0.3% of H_2_O_2_. Then we incubated the sections with rabbit antibodies anti caspase 3 (Catalog No. ab2057 33, dilated 1:100; Abcam), anti-NF-ĸB P65 (Catalog # PA5-27617, 1:100 dilution; Invitrogen), and PCNA (Catalog No. ab182858 dilated 1:500; Abcam). We incubated with goat anti-rabbit secondary antibody at room temperature using Dak, Aglint (catalog No. K 4003En TM system horse- reddish peroxidase label polymers). DAB kit was used to visualize the section for detection of brown color, and lastly, counterstaining it with Mayer’s hematoxylin [34]. The intensity of the immunostained area is expressed by the percentage of the definite positivity area in 8 High power fields examining 1000 cells [35].

### 2.9. Measurement of Cytokines in Testicular Tissue 

Inflammatory cytokines IL-6, IL-1, and TNFα were assayed using ELISA commercial kits with catalog numbers (Invitrogen, Cat number KHC3011, BMS224–2, EH2IL6, Thermo Fisher Scientific industry. Waltham, MA, USA) respectively, following the manufacturer’s instructions.

### 2.10. Analysis of Testicular Tissue and Sperm Ultra-Structure by Electron Microscope

Using a razor, we cut some of the right testes into pieces for fixation, then washed them with phosphate buffer and fixed them in osmium tetrachloride 1% rehydrating it by ascending concentration of alcohol, after that embedding it into an epoxy resin capsule and staining with toluidine blue to select the appropriate area. Using the diamond knife, we cut ultrathin sections on copper grids and stained them with uranyl acetate, followed by lead citrate [36]. Testicular tissue was photographed by electron microscope JEOL_JEM-100 SX (Jeol Limited, Tokyo. Japan) at Mansoura university, Egypt. To evaluate, the semen samples were centrifugated at 500× *g* for twenty minutes, and sperm pellets were claimed. Specimens were fixed in a solution composed of 2% paraformaldehyde, 2.5% buffered glutaraldehyde, and 0.1 M sodium phosphate overnight at 4 °C followed by fixation in tetrachloride 1% for an hour. Then dehydrated with alcohol. After the acetone evaporated, the specimens were retrieved. The membranes were then covered with gold and studied under a scanning electron microscope (JEOL JSM_6510 L, V). The microscope was used at 30 KV. Only the sample’s center regions were looked at (in one replicate, 100 sperm per specimen) and it was investigated how often broken heads and damage to the tail region occurred. 

### 2.11. RT-PCR Assessment

Our study used TRIzols Reagent (15596026, Life Technologies, Carlsbad, CA, USA) to collect total RNA from the testicular tissue. To calculate the purity and concentration of RNA, we used spectrophotometric methods at 260/280 nm. We used QuantiTect reverse transcription kit (Qiagen) to yield 1 µg of total RNA into single-strand paired DNA, following the manufacturer’s order. The primer sequences for Nrf2 and HO-1 genes, as well as the reference control (GAPDH) gene, are shown in (Table 2). SYBR green qRT-PCR performs real-time polymerase chain reaction: we started the denaturation following the stander procedure. Rotor-Gene Q (Qiagen) collects and automatically analyzes the data, which evaluates the housekeeping genes’ upper limit run rate. To achieve the comparative expression of Nrf2, HO-1, and GAPDH mRNA, we used the 2^−∆∆Ct^ technique.

### 2.12. Statistical Analysis 

We used GraphPad Prism software (version 9, O: GraphPad Prism software. La-Jolla, CA, USA). Values were reported as mean ± standard deviation. In our work, ANOVA in one direction with Tukey–Kramer analysis was applied. *p* < 0.05 was taken as significant. 

## 3. Results

### 3.1. The Outcome of Zamzam Water on Variations of Body and Testicular Induced by Administration of Gentamicin

There were differences between the group regarding body weight (Table 3). Concerning the weight of the testes, the control group revealed 3.1 ± 0.40, while gentamycin showed a significant reduction of 2.4 ± 0.38 (*p* < 0.01). At the same time, the drinking of Zamzam water in all the periods of the experiments significantly improved the weight of the testes (2.9 ± 0.47) (*p* < 0.05) in relation to gentamicin. Zamzam water preserved the mass of testicular tissues, decreasing its weight loss, most probably due to the antioxidant effect of Zamzam.

### 3.2. Ameliorating Outcome of Zamzam Water upon Gentamicin-Induced Oxidative Insult and Variation of Pituitary-Gonadal Axis

The effect of gentamycin on testicular tissues’ SOD, GPx CAT, and MDA (Table 4), treating rats with 100 mg gentamicin for continuous 6 days significantly (*p* < 0.001) elevated 36.48 ± 3.09 lipid peroxidation marker MDA in comparison to the control groups’ 9.5 ± 1.01. On the contrary, gentamicin treatment significantly (*p* < 0.001) decreased the tissue antioxidant enzymes GPx, SOD, and CAT levels (0.98 ± 0.12, 88.9 ± 7.9, 1.23 ± 0.27, respectively) in relation to the control group. In comparison, drinking Zamzam water with gentamicin treatment significantly (*p* < 0.001) improved the level of testicular tissue antioxidant enzymes GPx, SOD, and CAT (2.83 ± 0.42, 144.9 ± 9.35, and 3.06 ± 0.55, respectively) in relation to the gentamicin group. The level of hormonal changes of the Pituitary-gonadal axis during the treatment with gentamycin significantly (*p* < 0.001) decreased testosterone, FSH, and LH hormone levels (1.99 ± 0.33, 0.40 ± 0.06, and 1.14 ± 0.09, respectively) in comparison to the control group and group drinking Zamzam water significantly (*p* < 0.001 and *p* < 0.01) increased hormone level namely testosterone, FSH and LH in relation to gentamicin group. Zamzam water exhibits a powerful testicular antioxidant property with a concomitant positive regulatory effect on the pituitary–gonadal axis this may be explained by its antioxiant impact on genatmicin-induced ledyge cells’ oxidative insult upgrading the level of testosterone. 

### 3.3. Zamzam Water Impact upon Nrf2/Ho_1 Path in Testicular Tissue

**Figure 1:**
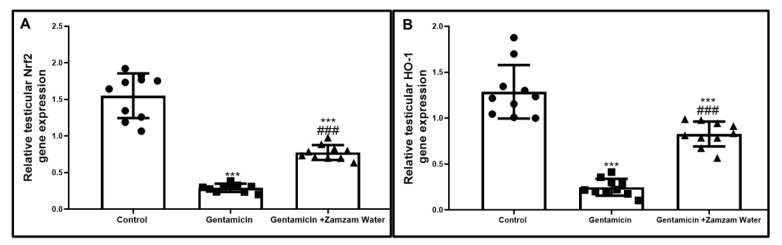
Effect of Zamzam water on gene expression; (**A**) Nrf2 and (**B**) its antioxidant H0-1. Values reported as mean ± standard deviation. ANOVA in one direction with Tukey post hoc analysis significant differences over the control group: *** *p* < 0.00. Significant versus the control group. The gentamicin group: ^###^ *p* < 0.00.

Gentamicin administration substantially (*p* < 0.001) downregulated nuclear prooxidant response gene *Nrf2* and its antioxidant *Ho_1* (0.292 ± 0.056, 0.247 ± 0.091) compared to the control group, Zamzam water cotreatment substantially *p* < 0.001 increased Nrf2 and HO-1 at the level of gene expression by 0.775 ± 0.308, 0.827 ± 0.135 in relation to gentamicin group (Figure 1), indicating the powerful antioxidant character of Zamzam water regarding its upregulatory effect on Nrf2/HO-1 pathway.

### 3.4. Zamzam Water Impact upon Spermatic Parameters

**Figure 2:**
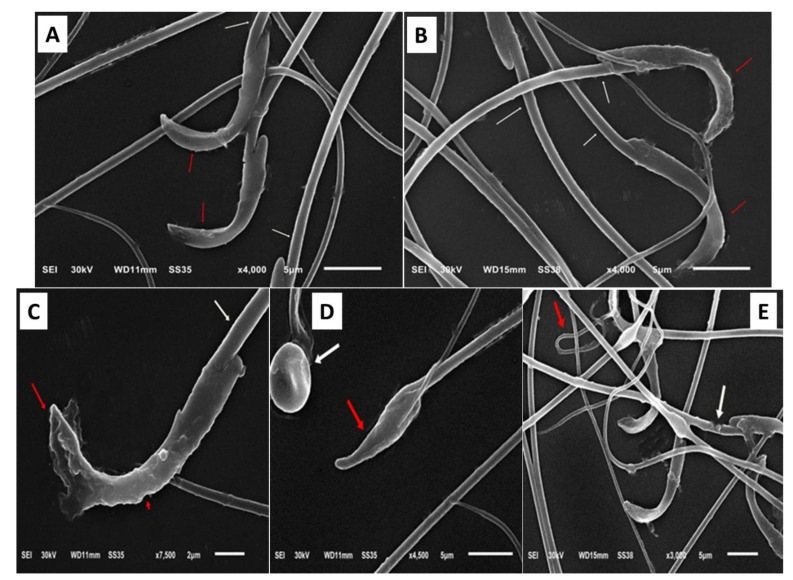
Representative images of scanning electron microscopy showing examples of sperm cell normal and abnormal. (**A**,**B**) normal sperm head (red arrow), normal middle piece (white arrow). (**C**–**E**) abnormal sperm; (**C**) damaged acrosomal and plasma membrane (red arrow), (**D**) small head (red arrow), and round head abnormalities (white arrow), (**E**) coiled tail (red arrow) and damaged middle piece (white arrow).

Gentamycin substantially *p* < 0.001 decreased the amount of sperm general motility and forward motility by 27.9 ± 5.62 and 12.6 ± 2.01, respectively, while the percentage of dead sperm and abnormal sperm increased to 46.7 ± 4.21 and 29.3 ± 2.75 compared with the control group (Table 5). Meanwhile, drinking Zamzam water significantly (*p* < 0.001) improved sperm parameters, increasing the amount of sperm general motility and forward motility while decreasing the percentage of dead sperm and abnormal sperm. The scanning electron microscope examination for semen in (Figure 2) revealed abnormal forms of the sperm in the form of damaged acrosomal and plasma membrane, small heads, round head abnormalities, and a coiled tail and damaged middle piece. These abnormal forms were significantly increased in gentamycin and decreased in Zamzam-treated rats. From the aforementioned, we can report the positive role of Zamzam water on spermatic parameters due to its antioxidant property.

### 3.5. Ameliorative Effect of Zamzam Water on Gentamicin-Induced Testicular Tissue Damage and Glycogen Store Depletion

**Figure 3:**
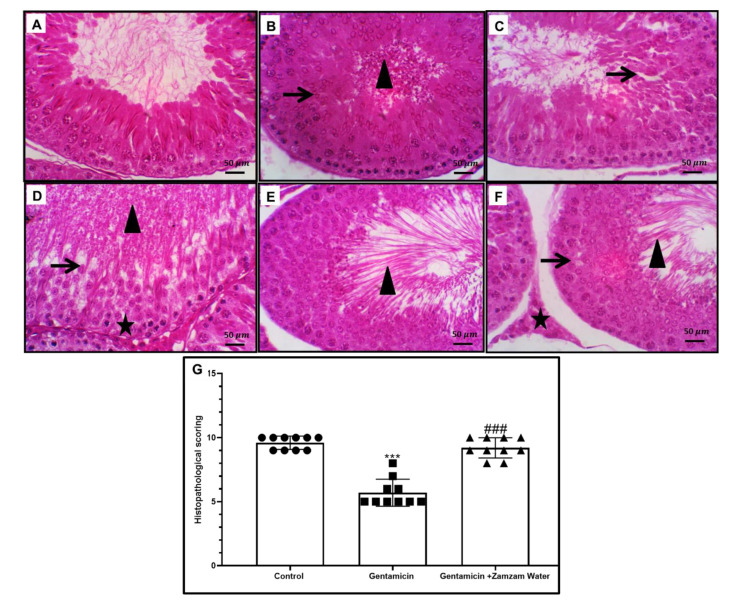
Representative photomicrographs of testis cross-section from the control and experimental groups. (**A**) The control groups displayed a compact and regular arrangement of spermatogenic cells and the presence of variable stages of maturation with many mature spermatozoa. (**B**,**C**) The gentamicin group showed distorted testicular architecture with numerous spermatogenic germ cell layers necrosis (mostly in spermatids and spermatozoa) (thin arrows), and intraluminal clumped sloughed germ cells admixed with necrotic sperm (arrowhead). (**D**) Gentamicin group showing testicular degenerative changes represented by severe spermatogenic vacuolation mostly in spermatids cells (thin arrow) with spermatogonial pyknosis (star) and marked eosinophilic necrotic debris closed the tubular lumen (arrowhead). (**E**,**F**) Zamzam group shows normal histological arrangement regular arrangement of spermatogenic cells and the presence of variable stages of maturation with a considerable number of mature spermatozoa, few, occasional vacuolated spermatogenic cells (thin arrow), and numerous intraluminal mature sperm cells (arrowheads). Leydig cells were normal (star). (**G**) Histogram of testicular histopathological scoring of different groups; values reported as mean ± standard deviation. ANOVA in one direction with Tukey post hoc analysis showing significant differences over the control group: *** *p* < 0.00, significant versus the control group. The gentamicin group: ^###^ *p* < 0.00. Image magnification 100× (**A**,**C**,**E**) ×400 (**B**,**D**,**F**) (**B**,**D**,**F**) bar 50.

**Figure 4:**
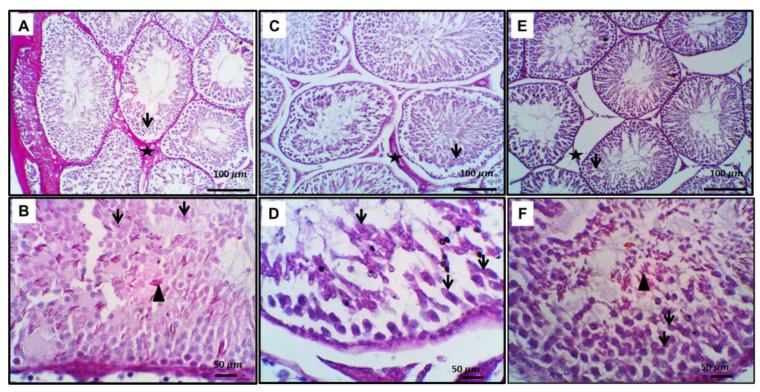
Control and experimental groups representative slides of periodic acid Schiff stain. (**A**,**B**) Control testis sections showed well-developed spermatogenesis, positivity in Leydig cells (star) inset, showed round spermatid with a normal acrosomal cap (arrowhead), and elongated spermatid (arrows). (**C**,**D**) Shows marked substandard sperm production with aberrant acrosomal cap (arrows) and deformed elongated spermatids with intense intracytoplasmic positivity. (**E**,**F**) Zamzam group showed well-developed spermatogenesis, positivity inset, showed round spermatid with a normal acrosomal cap (arrowhead), and elongated spermatid (arrowheads). Image magnification 100× (**A**,**C**,**E**) ×400 (**B**,**D**,**F**) (**B**,**D**,**F**) bar 50.

H&E stain showed normal testicular tissue in the control group (Figure 3). Treatment with gentamicin exhibits alternation in the testicular morphology in the form of severe spermatogenic vacuolation with spermatogonial pyknosis, with necrosis of most spermatogenic germ cell layers, especially in spermatids and spermatozoa, and intraluminal clumped sloughed germ cells admixed with necrotic sperm with a Johnsen’s score of 5. Treatment of the gentamicin group with Zamzam water increased the Johnsen’s score to 9 with a normal histological arrangement, S. tubule variable stages of maturation across the seminiferous tubule, few, occasional vacuolated spermatogenic cells, and numerous intraluminal mature sperm cells. Leydig cells appeared normal. Glycogen content of testicular tissue and microscopic examination of testicular tissue stained with PAS on control testis sections showed normal spermatogenesis and PAS-positive Leydig cells. Inset: here round and elongated spermatids. Acrosomal cap showed PAS positivity, while gentamicin-treated rats showed a significant decrease in glycogen content in the form of marked arrested maturation with a deformed acrosomal cap and altered elongated spermatids with intense intracytoplasmic positivity. On the other hand, gentamicin cotreated with Zamzam water showed significant improvement in glycogen content, normal spermatogenesis, and PAS-positive Leydig cells. Inset: here round and elongated spermatids. The acrosomal cap showed PAS positivity (Figure 4). From all of these findings, we can conclude that drinking Zamzam water markedly improves the histological structure of testicular tissues alleviating gentamicin-induced testicular weight loss.

### 3.6. Impact of Zamzam Water on Gentamicin-Induced Testicular Ultrastructural Changes

**Figure 5:**
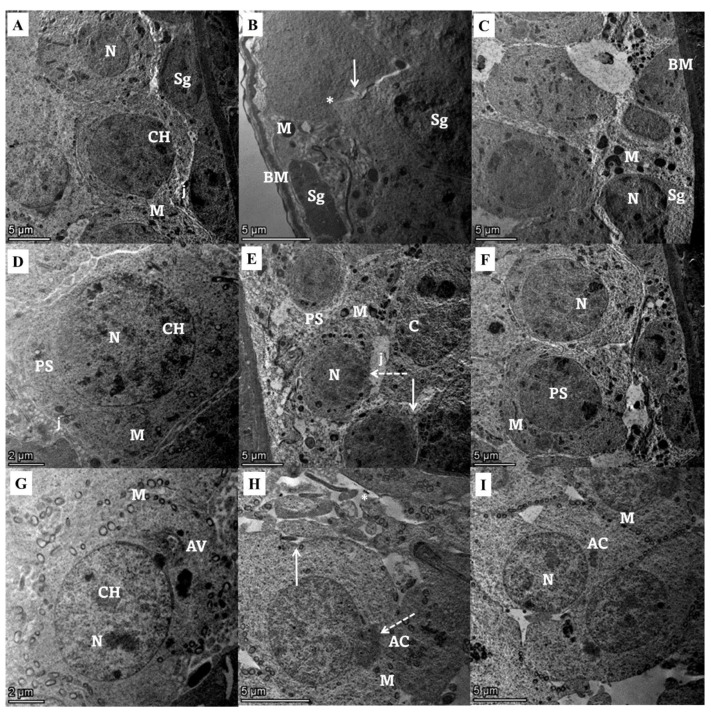
Transmission electron micrograph of seminiferous tubular lining cells from different experimental groups. (1) Spermatogonia cells (**A**) control group shows a normal arrangement of spermatogenic cells, including numerous spermatogonia (Sg) with normal nucleus (N) and peripherally arranged mitochondria (M), and chromatoid body (CH) and cells, showing Normal junction (j) in between. (**B**) Gentamicin group showing irregular basement membrane (BM), spermatogonia (Sg) with damaged nuclear membrane and compact Electron dense granule Swollen mitochondria (M), and irregular cell outline with loss of tight junction in between increasing intercellular gap (arrow). (**C**) Zamzam treated group showed normal basement membrane (BM), minimally enlarged spermatogonia (Sg) with nearly normal nucleus (N), and granular cytoplasm containing many small mitochondria (M); it appears as control with minimal loss of the epithelial tight junction. (2) Primary spermatocytes (**D**) Control group showing normal primary spermatocyte (PS) with prominent well-defined euchromatic nucleus (N), chromatoid body (CH), and fine cytoplasm containing many normal mitochondria (M) cells with tight junction (j). (**E**) Gentamicin group showing vacuolated primary spermatocyte with irregular cell outline (arrow) with focal nuclear membrane damage (dotted arrow) besides numerous enlarged mitochondria (M) and wide intercellular gap (arrow). (**F**) Zamzam treated group showed slightly enlarged spermatocytes with nearly normal oval shape nucleus (N), regular nuclear membrane, and many normal mitochondria. 3) Spermatids (**G**) control group showing normal spermatids with its characteristic complete acrosomal cap (AC) and acrosomal vesicles (AV). Its nucleus (N) is euchromatic with fine granular chromatin (CH) and peripherally arranged vesicular mitochondria (M). (**H**) Gentamycin group showing spermatids with irregular cell outline (shrunken) with membrane rupture (Arrow) and local distribution of some cytoplasmic irregular compact mitochondria (M) beside irregularly shaped (AC) to detached acrosomal cap (dotted arrow) multivaculation in intercellular space increasing intracellular gaps (*). (**I**) Zamzam treated group showing well-developed and activated spermatids with definite cell outlines and normally arranged mitochondria (M), cells show typical cell to cell junction decreasing intracellular spaces. (TEM, bar, 5 µm **A**,**B**,**C**,**E**,**F**,**I**. J.TEM, bar, 2 µm **D**,**J**).

Ultrastructural examination of seminiferous tubular lining cells from different experimental groups showed a typical ultrastructural picture of spermatogenic, primary spermatocytes, and spermatid cells, while the group treated with gentamycin showed spermatogenic cells with irregular basement membrane with damage nuclear membrane and Swollen mitochondria, with loss of the tight junction in between the increasing intercellular gap. Primary spermatocytes with focal nuclear membrane damage beside numerous enlarged mitochondria and wide intercellular gap, and spermatids with membrane ruptured cell membrane with irregularly shaped to detached acrosomal cap with the enhancement of intracellular gaps. Marked improvement in the ultrastructural shape of seminiferous tubular cells with the treatment with Zamzam water in the form of spermatogenic cells with the normal basement membrane, nucleus, mitochondria, and minimal loss of the epithelial tight junction, slightly enlarged primary spermatocyte with nearly normal oval shape nucleus, regular nuclear membrane and many normal mitochondria, and spermatids with definite cell outline and typical cell to cell junction decreasing intracellular spaces (Figure 5).

### 3.7. Ameliorative Effect of Zamzam Water on Gentamicin-Induced Testicular Tissue Inflammation

**Figure 6:**
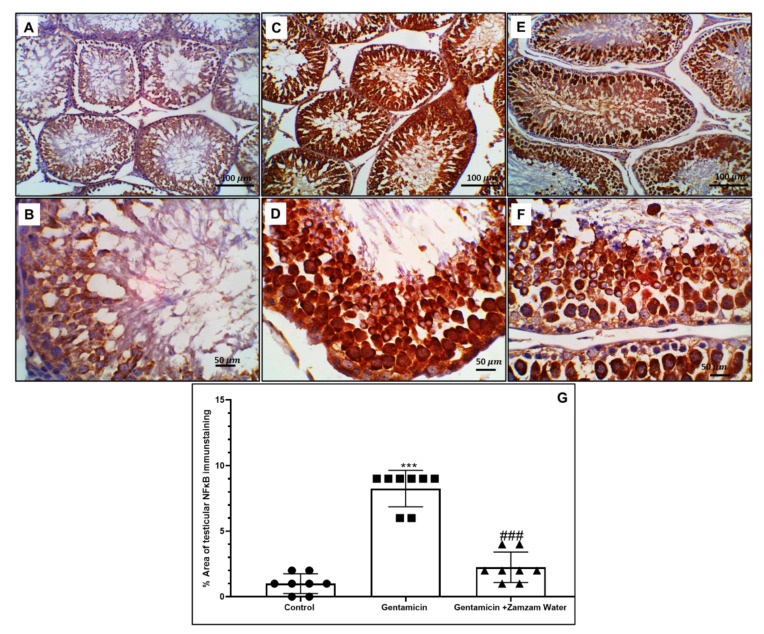
Typical immunohistochemistry staining of NF-B expression in experimental and control testicle groups. (**A**) Control group showing diffuse minimal expression of NF-κB in spermatogenic cells and interstitial Leydig cells, (**B**) higher power showing few cytoplasmic expressions of NF-κB. (**C**) Gentamicin group showing the diffuse, extensive expression of NF-κB in spermatogenic cells and interstitial Leydig cells. (**D**) higher power showing intense cytoplasmic and nuclear expression in spermatocytes with more expression in the acrosomal cap of spermatids. (**E**) The Zamzam group showing a moderate decrease in NF-κB expression in seminiferous tubules with few expressions in interstitial Leydig cells. (**F**) The Zamzam group higher power showing the localization of NF-κB within spermatogenic cells with scant expression in spermatogonia, less cytoplasmic expression in spermatocytes and more expression in the acrosomal cap of spermatids without nuclear expression. (**G**) Testicular NF-κB immunostaining histogram percent area; values reported as mean ± standard deviation. ANOVA in one direction with Tukey post hoc analysis showing significant differences over the control group: *** *p* < 0.00, significant versus the control group. The gentamicin group: ^###^ *p* < 0.00, image magnification 100× (**A**,**C**,**E**) ×400 (**B**,**D**,**F**) (**B**,**D**,**F**) bar 50.

**Figure 7:**
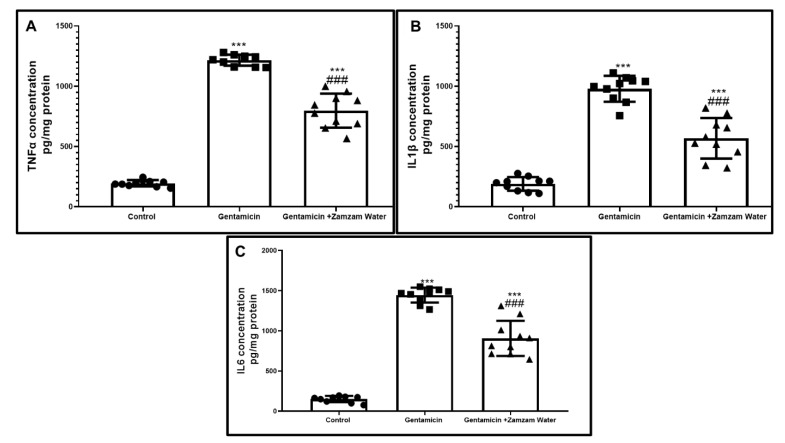
Effect of Zamzam water treatment on proinflammatory markers by ELIZA: (**A**) TNFα, (**B**) IL1β, and (**C**) IL-6 in different groups of rats. Values reported as mean ± standard deviation. ANOVA in one direction with Tukey post hoc analysis showing significant differences over the control group: *** *p* < 0.00, significant versus the control group. The gentamicin group: ^###^ *p* < 0.00.

Immunohistochemical examination of testicular tissue with Nf-κB significantly (*p* < 0.001) increased in gentamicin assemblage in relation to the control assemblage, while Zamzam water cotreatment significantly (*p* < 0.001) downregulated the immunoexpression in relation to gentamicin group (Figure 6), as a result of stimulation of Nf-κB nuclear transcriptional factor, flu of inflammatory cytokines TNFα, IL-1β, IL-6 significantly (*p* < 0.001) 1216 ± 45.8, 979 ± 107.8, and 1445 ± 92.5 upregulated, respectively, in comparison to the control group. On the contrary, the treatment of the gentamicin group with Zamzam water significantly (*p* < 0.001) 798 ± 141.2, 568 ± 168, and 809 ± 218.6 downregulated, respectively, in comparison to the gentamicin group (Figure 7), indicating the anti-inflammatory character of Zamzam water. These results confirm the anti-inflammatory role of Zamzam water against gentamicin-induced testicular inflammation.

### 3.8. Zamzam Water Mitigates Gentamicin-Induced Testicular Apoptosis

**Figure 8:**
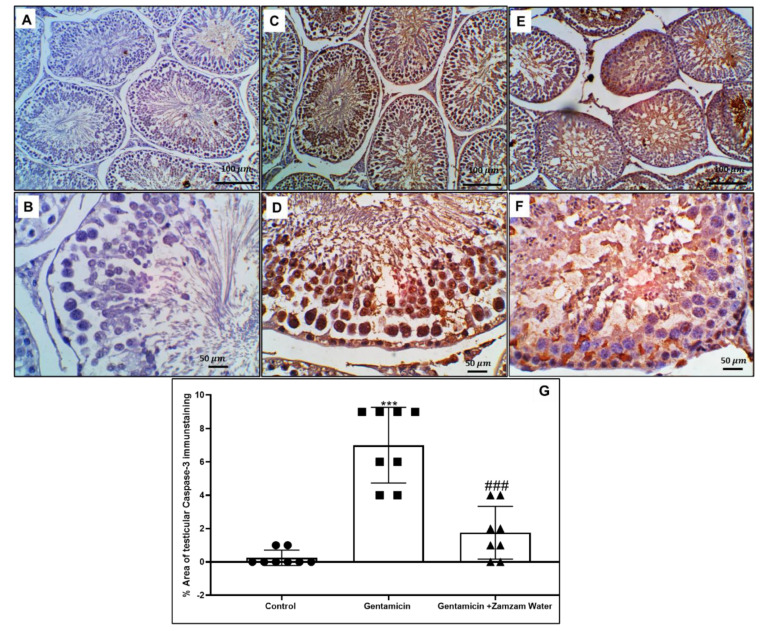
Illustrative immunohistochemistry stain of the expression of caspase-3 in the testes of the experimental and control groups. (**A**) In spermatogenic cells, the control group demonstrated no expression of caspase-3, and (**B**) the high power of the control group demonstrated no expression of caspase-3 in seminiferous tubular spermatogenic cells or interstitial Leydig cells. (**C**) The gentamicin group displayed varied spermatogenic germ cells with diffuse, marked, or strong caspase-3 expression, and in interstitial Leydig cells, (**D**) higher power showing intense cytoplasmic and nuclear expression of caspase-3 in different cells of the spermatogenic epithelium. (**E**) The Zamzam group showed less expression of caspase-3 within spermatogenic and interstitial Leydig cells. (**F**) Higher power on seminiferous tubules of the Zamzam group showing scanty cytoplasmic expression in spermatogonia and primary spermatocytes with more cytoplasmic expression within spermatozoa. (**G**) Testicular caspase-3 immunostaining histogram percent area; values reported as mean ± standard deviation. ANOVA in one direction with Tukey post hoc analysis showing significant differences over the control group: *** *p* < 0.00, significant versus the control group. The gentamicin group: ^###^ *p* < 0.00, image magnification 100× (**A**,**C**,**E**) ×400 (**B**,**D**,**F**) (**B**,**D**,**F**) bar 50.

Administration of 100 mg/kg/day intraperitoneally for six days (*p* < 0.001) increased apoptotic caspase-3 in different spermatogenic epithelium cells in relation to the control group, contrasted with cotreatment use of the gentamicin assemblage with Zamzam water significantly (*p* < 0.001) decreasing the caspase-3 apoptotic spermatogenic germ cells in relation to the gentamicin group (Figure 8). This indicates the antiapoptotic effect of Zamzam water on spermatogenic cells as a normal result of its antioxidant and anti-inflammatory character.

### 3.9. Ameliorative Effect of Zamzam Water on Gentamicin Impaired Spermatogenesis

**Figure 9:**
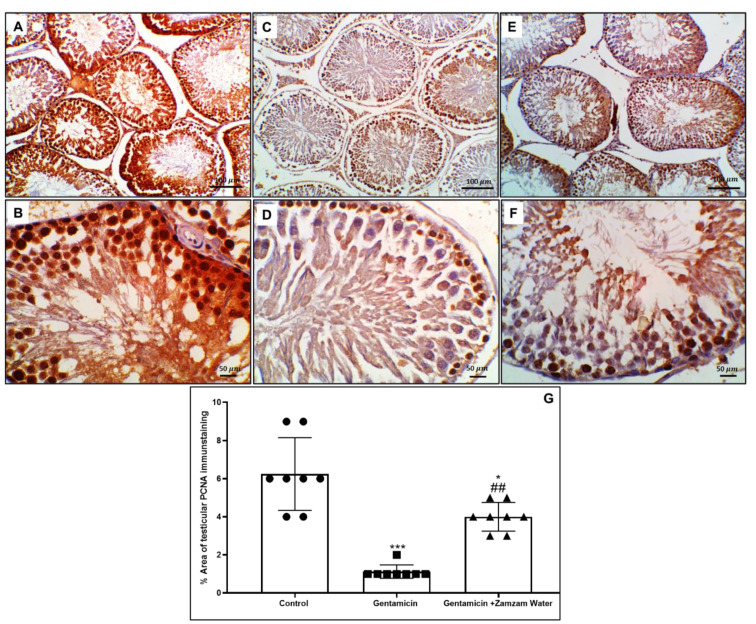
Illustrative immunohistochemistry stain of proliferating cells nuclear antigen (PCNA) in the testes of the experimental and control groups (**A**) the control group showed strict nuclear manifestation of PCNA, especially the spermatogonial stem cells in a few testicular tubules. (**B**) higher power of the control group showing more nuclear expression in spermatogonial cells with faint cytoplasmic and less nuclear expression in spermatocytes and spermatozoa. (**C**) Gentamicin assemblage showed diffuse, significant expression PCNA staining in spermatogenic cells and interstitial Leydig cells. (**D**) Higher power on seminiferous tubules of cis group showing PCNA was localized in most tubular cell layers with intense expression in spermatogonia and varied nuclear to cytoplasmic expression in spermatocytes, spermatids. (**E**) The Zamzam group showed a marked reduction in PCNA expression in seminiferous tubules with scant expression in interstitial Leydig cells. (**F**) higher power on seminiferous tubules showing more localization of PCNA in spermatogonial cells with faint strictly nuclear expression mostly in secondary spermatocytes and few spermatids and spermatozoa, some intense cytoplasmic to nuclear expression in spermatids and spermatozoa were seen toward the lumen of seminiferous tubules. (**G**) Testicular PCNA immunostaining histogram percent area; values reported as mean ± standard deviation. ANOVA in one direction with Tukey post hoc analysis showing significant differences over the control group: *** *p* < 0.00, * *p* < 0.05 Significant versus the control group. The gentamicin group:, ## *p* < 0.01, image magnification 100× (**A**,**C**,**E**) ×400 (**B**,**D**,**F**) (**B**,**D**,**F**) bar 50.

PCNA immunoexpression in the seminiferous tubules was considered an indicator for spermatogenesis; the immunoexpression of PCNA in control and Zamzam water were significantly (*p* < 0.00) and (*p* < 0.01) enhanced in relation to the gentamicin group (Figure 9), this indicates the good impact of Zamzam water on the spermatogenesis process as a result to its antioxidant character.

## 4. Discussion

Gentamicin was considered one of the common antibiotics that affect the process of spermatogenesis and functions of the sperm [37,38,39,40,41]. Several studies were performed on agents that can scavenge ROS production and their protective effects on gentamicin-induced reproductive organ damage [42,43,44]. In this study, we assessed the ameliorative role of Zamzam water as an example of common alkaline water against gentamicin-induced testicular damage. The findings of the current investigation showed that intraperitoneal injection of rats with gentamicin 100 mg/kg for six days induced severe oxidative stress, marked reduction in testicular weight, affection of sperm parameters, morphological alterations in seminiferous tubules with different cell lines, testicular inflammation, and impaired spermatogenesis process. These results agree with Narayana, [41], who reported that gentamicin-induced oxidative stress plays a leading cause in testicular toxicity and sperm affection. Moreover, Zamzam water has been reported to exert an antioxidant and anti-inflammatory effect against nephrotoxicity and hepatotoxicity induced by gentamicin [45]. In our study, the body weight of the rats is not significantly affected by gentamicin administration, however, the testicular weight was significantly (*p* < 0.01) reduced., The testicular weight is mainly dependent on the seminiferous tubule’s contents from germ cells and spermatozoa [46]. It decreases primarily due to the downregulation of testosterone levels with subsequent inhibition of the spermatogenesis process [47]. Testosterone level downregulation can be explained by gentamicin-induced toxicity to ledyge cells as a consequence of free radicals’ overproduction, resulting in impairment of spermatogenesis, as the deficiency of testosterone arrested it at the miosis stage [48] and affected the attachment of Sertoli cells to spermatid leading to its premature sloughing in the seminiferous tubular lumen [49,50]. Regarding the inhibitory effect of gentamicin on the pituitary-gonadal axis, it leads to impairment in steroidogenesis with a drop in testosterone level [51]. Side by side with the inhibitory effect of gentamicin on testosterone levels, it significantly (*p* < 0.001) upregulated FSH and LH hormonal levels, this result is consistent with the previous work done by Elsawah et al. [52]. The elevation in FSH and LH hormonal levels may result from the decrease in the inhibitory feedback of testosterone. 

In this research, testicular weight reduction in the gentamicin group was associated with alternation in histological and ultrastructural with severe spermatogenic vacuolation and pyknosis, with necrosis in spermatids and spermatozoa, and intraluminal clumped sloughed germ cells. These histopathological findings with subsequent loss of spermatozoa coincide with those of Narayana, [41] and Elsawah et al. [52]. The depletion of testicular glycogen content appeared as a weak PAS-positive reaction. Some authors explained this as the damaged cells’ loss of the capacity to metabolize the glycogen and its storage [53]. At the level of the ultrastructural study, gentamicin administration at 100 mg/kg for six days induced seminiferous different cells destruction in the form of an irregular basement membrane with the damaged nuclear membrane and swollen mitochondria, with loss of tight junction in between increasing intercellular gap with a detachment of spermatid acrosomal cap. These findings are in concurrence with Khaki et al. [54], who reported that gentamicin treatment caused testicular ultrastructural damage with mitochondria with lost cristae and lysosomes seen more in the cytoplasm of Sertoli cells. Drinking Zamzam water significantly (*p* < 0.05) improved testicular weight; this result is explained by two different mechanisms. First, its modulating effect on the pituitary-gonadal axis by significantly (*p* < 0.01) upregulating the level of testosterone level with the downregulation of FSH, and LH hormone improving the spermatogenesis process, and the second one is through its antioxidant property. The positive impact of Zamzam water on testicular weight matches with its modulatory effect on the morphological structure of the testes in the form of maturation of spermatogenic cells in the seminiferous tubules, with few vacuolated spermatogenic cells, abundant intraluminal mature sperm cells, normal interstitial space with apparently normal Leydig cells, increasing the glycogen content, and marked improvement in the ultrastructural shape of seminiferous tubular cells in the form of the normal basement membrane, nucleus, mitochondria, and minimal loss of the epithelial tight junction. This study revealed that treatment with gentamicin significantly (*p* < 0.001) altered the spermatic parameters as it reduced the motility of sperm and its progressive motility, increased the percentage of dead sperm and abnormal forms of the sperm either in the head, middle piece, or in tail pieces in relation to control rats. There is a considerable correlation between the motility and viability of the sperm, as the nonviable sperm loses the motility capacity. These ominous signs can be attributed to its inhibitory effect on testosterone with impaired epididymis maturation, which can also be explained by gentamicin-induced oxidative stress [55]. The toxicity of gentamicin on the spermatic parameter coincides with the study reported by Kim et al. [5]. Coadministration of Zamzam water with gentamicin significantly (*p* < 0.001) improved the sperm parameter and motility, decreasing the percentage of dead sperm and abnormal forms, which can be explained by its scavenging power on oxygen free radicals.

Moreover, in the current work, gentamicin administration markedly (*p* < 0.001) decreased PCNA immunoexpression compared to the control group. PCNA is considered an indicator for spermatogenic cell proliferation, DNA replication, and its affection means spermatogenesis arrest. Our finding coincides with that of Meligy et al. [56], who reported that gentamicin administration significantly reduced the PCNA immune positive cardiac cells in gentamicin-induced cardiac degeneration in comparison to control group. This can be explained by the accumulation of ROS, which has been suggested to induce DNA fragmentation [57]. On the other hand, drinking Zamzam water significantly (*p* < 0.01) elevated PCNA immunoexpression compared to gentamicin. This may be due to its antioxidant character. In our study, administration of gentamicin most probably generates a gush of free oxygen radicals which consumes the invitro protective antioxidant enzymes producing severe oxidative stress with significant (*p* < 0.001) elevation of the lipid peroxidation maker MDA, and downregulation of the antioxidant enzymes SOD, CAT, and GPx. SOD is considered the first line of defense against oxidative stress by its dismutation of free radicals. Gentamicin-induced reduction in the action of the catalase enzyme leads to impairment in eliminating hydrogen peroxide [58], and the reduction in the contents of glutathione affects the activity of GPx enzyme. CAT and GPx have a principal role in preventing SOD enzyme inactivation from hydrogen peroxides; mutually, SOD protects the CAT and GPx enzymes from hydrogen peroxides inhibition. In concurrence with the study of Meseguer et al. [57], gentamicin-induced oxidative stress. ROS exhibits severe damage to the spermatozoa and other membrane cytoplasmic organelles through its peroxidation to proteins, phospholipids, and nucleotides affecting the motility of the sperm [59], in addition to consuming the defense antioxidant enzymes [60]. Zamzam water with gentamicin has strong antioxidant properties, scavenging free radicals by significantly (*p* < 0.001) reducing MDA marker and upgrading the antioxidant enzyme level CAT, SOD. and GPx. These results are in line with those of Saif et al. [61], who reported that treatment of carbon tetrachloride-induced liver damage with Zamzam water significantly decreased hepatic lipid peroxidation marker MDA, increased hepatic SOD, CAT, and GPx enzymes with upregulation to the level of glutathione. Also, Zamzam water decreased the level of kidney MDA, and elevated glutathione levels in a diabetic-induced nephropathy in vitro and in vivo study carried out by El Maleky et al. [62]. Additionally, Sayed et al. [45] stated that pretreatment of mice affected by hepatotoxicity and nephrotoxicity caused by gentamicin with Zamzam water resulted in a decrease in MDA level and an increase in CAT activity. The strong antioxidant character of Zamzam water may be owed to its content of several antioxidant minerals like selenium, magnesium, strontium, and others in alkaline PH nature [23,63].

Along with the scavenging effect of Zamzam water on ROS formation, it exhibits a significant (*p* < 0.001) upregulation to *Nrf2/HO-1* pathway compared to the gentamicin group. Our results revealed significant (*p* < 0.001) downregulation in testicular *Nrf2/HO-1* gene expression in the gentamicin group in relation to control rats. This result coincides with Althunibat et al. [64], who reported that gentamicin administration downregulated immunoexpression of Nrf2 and HO-1 in a rat model of gentamicin-induced nephrotoxicity. Nrf2 is considered a redox-sensitive transcription factor that is sequestered by Keap-1 in the cytoplasm. After its activation by exposure to ROS, it will be translocated to the nucleus attaching to antioxidant-releasing elements stimulating antioxidant genes such as HO-1 [65]. As far as the author’s know, we are the first to examine the upregulating effect of Zamzam water on the Nrf2/HO-1 route and its apparent effect on the testicular battle against gentamicin toxicity. In our existing study, gentamicin administration significantly (*p* < 0.001) upregulated Nf-κB immunoexpression with subsequent enhancement in inflammatory markers IL-6. IL-1 and tumor necrosis factor by ELISA in relation to control rats, while treatment with Zamzam water significantly (*p* < 0.001) decreased immunoexpression of Nf-κB with inflammatory flu from cytokines in comparison to the gentamicin group. These findings coincide with the study done by Moni et al. [66], who reported that Zamzam water possesses a wound-healing property through its anti-inflammatory effect by downregulating inflammatory cytokines TNFα, IL-1β, and IL-6. Zamzam water’s effect in preventing inflammation can be explained by its content of zinc at the alkaline pH, which offers an anti-inflammatory character, also may be explained by [67,68], who suggested its ability to regulate IL-1β due to its content of arsenic as the arsenic associated with inhibition of Th1 and Th2 cytokines. One mechanism that impairs the process of spermatogenesis is apoptosis, as the oxidative insult stimulates the apoptotic intrinsic pathway by elevating Bax gene and depressing BCL-2 gene expression with a subsequent damaging mitochondrial membrane [69]. Consequently, the cytoplasmic release of mitochondrial membrane cytochrome c with subsequent activation to caspases cascade, including caspase-3, which stimulates DNase, which damages DNA [70], leading to apoptosis of the germ cells. In our work, gentamicin substantially (*p* < 0.01) increased caspase-3 immunoexpression in comparison to the control group. In contrast, Zamzam water substantially (*p* < 0.01), decreased Caspase-3 immunoexpression in comparison to the gentamicin group. The finding is consistent with El Maleky et al. [62], who reported its renoprotective against STZ-induced renal impairment through its downregulation to Caspase-3 immunoexpression, indicating its antiapoptotic property. The antiapoptotic effect of Zamzam water can be explained by its alkaline character, which reduces oxidative insult. In addition, Zamzam water zinc decreased the protein expression of the following caspases [3,7,8 and 9] in addition to cytochrome c, Bax, and Apaf- [71].

## 5. Conclusions

Zamzam water abrogated gentamicin-induced testicular toxicity; this was demonstrated by the restoration of testicular parameters, spermatogenesis, testicular histological architecture, and regulation of the pituitary–gonadal axis. The palliative effect of Zamzam water may be attributed to its distinctive antioxidant, anti-inflammatory, and antiapoptotic actions. However, future studies are warranted to gain deep insight into the role of Zamzam water on autophagy and apoptosis crosstalk in testicular damage caused by gentamicin. In addition, it is imperative to extend the study to human subjects to ascertain our findings and to evaluate the application of Zamzam water in patients suffering from infertility problems associated with gentamicin therapy.

## Figures and Tables

**Table 1:** Johnsen’s criteria for spermatogenesis scoring.

10	Complete spermatogenesis with many mature spermatozoa
9	Many spermatozoa, with a disorganized germinal epithelium that showed sloughing into lumen
8	Presence of few spermatozoa (<5 to 10/seminiferous tubule)
7	Absence of spermatozoa, but many spermatids are present
6	Absence of spermatozoa, with few spermatids (<5/seminiferous tubule)
5	Absence of spermatozoa, and spermatid, with the presence of several spermatocytes
4	Absence of spermatozoa, and spermatid, with the presence of few spermatocytes
3	Spermatogonia are the only cell present
2	Sertoli cells only present with absence of germ cells
1	No cells visualized in the tubular section

**Table 2:** Shows the primer’s sequence.

	Forward Sequence	Reverse Sequence	Gene Accession Number
Nrf2	AGGACATGGAGCAAGTTTGG	TTGCCCTAAGCTCATCTCGT	NM_031789.2
HO-1	TCAGGTGTCCAGAGAAGGCTTT	CTCTTCCAGGGCCGTGTAGA	NM_012580.2
GAPDH	CCTTCTCCATGGTGGTGAAGA	CACCATCTTCCAGGAGCGAG	NM_001394060.2

**Table 3:** Effect of Zamzam water on body and testes weights.

	Control Group	Gentamicin Group	Gentamicin + Zamzam Water
Initial body weight (gm)	142.2 ± 10.2	142.7 ± 12.05	146.9 ± 13.4
Final body weight (gm)	205 ± 12.57	189.7 ± 14.52	202.3 ± 16.6
Weight of the testes (gm)	3.1 ± 0.41	2.42 ± 0.38 **	2.9 ± 0.47 ^#^

Values reported as mean ± standard deviation. ANOVA in one direction with Tukey’s post hoc analysis showed significant differences over the control group: ** *p* < 0.01, significant versus the control group. The gentamicin group: ^#^  *p* < 0.05 (n = 10).

**Table 4:** Effect of Zamzam water on biochemical parameters.

Group	Testicular Tissue				Serum		
	GPx μmol/g	SOD U/g	MDA nmol/g	CAT U/g	Testosterone (nmol/L)	FSH (mIU/mL)	LH (mIU/mL)
Control group	3.02 ± 0.23	187.6 ± 8.05	9.5 ± 1.01	4.14 ± 0.22	5.01 ± 0.53	0.93 ± 0.07	2.37 ± 0.18
Gentamicin group	0.98 ± 0.12 ***	88.9 ± 7.9 ***	36.48 ± 3.09 ***	1.23 ± 0.27 ***	1.99 ± 0.33 ***	0.40 ± 0.06 ***	1.14 ± 0.09 ***
Gentamicin + Zamzam water group	2.83 ± 0.42 ^###^	144.9 ± 9.35 ^###^	25.49 ± 2.77 ^###^	3.06 ± 0.55 ^###^	2.83 ± 0.26 ^##^ 0.0004	0.77 ± 0.06 ^###^	1.68 ± 0.13 ^###^

Values reported as mean ± standard deviation. ANOVA in one direction with Tukey post hoc analysis showed significant differences over the control group: *** *p* < 0.00, significant versus the control group. The gentamicin group: ^###^ *p* < 0.00, ^##^ *p* < 0.01, (n = 10).

**Table 5:** Effect of Zamzam water on spermatic parameters.

	Percentage of Sperm Motility %	Percentage of Progressive Motility %	Percentage of Dead Sperm %	Percentage of Abnormal Sperm %
Control group	68.3 ± 2.49	42.8 ± 2.15	20.0 ± 1.94	12.1 ± 2.13
Gentamicin group	27.9 ± 5.62 ***	12.6 ± 2.01 ***	46.7 ± 4.21 ***	29.3 ± 2.75 ***
Gentamicin + Zamzam water group	49.8 ± 4.06 ^###^	18.1 ± 4.86 ^#^ *p* = 0.0024	29.4 ± 2.87 ^###^	21.5 ± 2.46 ^###^

Values reported as mean ± standard deviation. ANOVA in one direction with Tukey post hoc analysis showed significant differences over the control group: *** *p* < 0.00, Significant versus the control group. The gentamicin group: ^###^ *p* < 0.00, ^#^ *p* < 0.05 (n = 10).

## Data Availability

The data that support this research will be shared upon reasonable request to the corresponding authors.

## Abbreviations

Apaf-1	apoptotic protease activating factor 1
Bax	Bcl-2-associated protein
BCL-2	B-cell lymphoma 2
CAT	catalase
ELISA	enzyme-linked immunosorbent assay
FSH	follicle-stimulating hormone
GAPDH	glyceraldehyde-3-phosphate dehydrogenase
GPx	glutathione peroxidase
IL-1β	interleukin-1β
IL-6	interleukin 6
LH	luteinizing hormone
NF-κB	nuclear factors kappa-β
Nrf2/HO-1	the nuclear factor erythroid 2-related factor transcription factor/hemoxygenase 1
HbA1c	hemoglobin A1C
PAS	periodic-acid-Schiff
PCNA	proliferating cell nuclear antigen
ROS	reactive oxygen species
SOD	superoxide dismutase
STZ	streptozotocin
Th1	type 1 T helper (Th1) cells
Th2	type 2 T helper (Th1) cells
TNF-α	tumor necrosis factor alpha
